# Biochemical Characterization of APPL Endosomes: The Role of Annexin A2 in APPL Membrane Recruitment

**DOI:** 10.1111/j.1600-0854.2011.01226.x

**Published:** 2011-07-03

**Authors:** Anna Urbanska, Lukasz Sadowski, Yannis Kalaidzidis, Marta Miaczynska

**Affiliations:** 1 International Institute of Molecular and Cell Biology, Laboratory of Cell Biology, International Institute of Molecular and Cell Biology4 Ks. Trojdena Street, 02-109, Warsaw, Poland; 2 Max Planck Institute of Molecular Cell Biology and GeneticsPfotenhauerstr. 108, 01307, Dresden, Germany

**Keywords:** Annexin A2, APPL1, APPL2, endocytosis, endosomes

## Abstract

APPL endosomes are a recently identified subpopulation of early endosomes characterized by the presence of two homologous Rab5 effector proteins APPL1 and APPL2. They exhibit only limited colocalization with EEA1, another Rab5 effector and a marker of the canonical early endosomes. Although APPL endosomes appear to play important roles in cargo trafficking and signal transduction, their protein composition and biochemical properties remain largely unknown. Here we employed membrane fractionation methods to characterize APPL endosomes biochemically. We demonstrate that they represent heterogeneous membrane structures which can be discriminated from the canonical EEA1-positive early endosomes by their partly different physical properties and a distinct migration pattern in the continuous density gradients. In search for other potential markers of APPL endosomes we identified Annexin A2 as an interacting partner of both APPL1 and APPL2. Annexin A2 is a Ca^2+^ and phosphatidylinositol 4,5-bisphosphate binding protein, previously implicated in several endocytic steps. We show that Annexin A2 co-fractionates and colocalizes with APPL endosomes. Moreover, silencing of its expression causes solubilization of APPL2 from endosomes. Although Annexin A2 is not an exclusive marker of APPL endosomes, our data suggest that it has an important function in membrane recruitment of APPL proteins, acting in parallel to Rab5.

Endocytosis involves internalization of macromolecules from the cell surface into internal membrane compartments (1). Various cargo molecules use different entry portals into cells which include well-characterized clathrin-mediated endocytosis and other clathrin-independent internalization pathways (2). Following uptake, cargo molecules are sorted within the endosomal system and follow one of the transport routes, resulting in recycling to the plasma membrane, degradation in the lysosome or retrieval to the Golgi apparatus.

Within the cell, one of the main cargo sorting stations are early endosomes which harbor small GTPase Rab5 as their principal marker (3,4). Rab5 localizes also to clathrin-coated vesicles (CCVs) and is a master regulator of early steps of endocytosis (3). It operates via a number of effector proteins which ensure tethering, fusion and cytoskeleton-mediated motility of early endosomes (5–11). One of the key Rab5 effectors is early endosome antigen EEA1 (5,12). It localizes to the canonical Rab5-positive early endosomes enriched in phosphatidylinositol 3-phosphate [PI(3)P] and is considered a specific marker for this compartment (13). However, in addition to the canonical EEA1-positive endosomes, recent reports demonstrated existence of Rab5-positive, PI(3)P-negative early endosomes which harbor one or both homologous adaptor proteins APPL1 and APPL2 (14–16). Like EEA1, APPL proteins act as effectors of Rab5 and are recruited to the endosomal membrane via interaction with an active form of this GTPase (15). Both APPL proteins can form homo- and heterodimers (17–20). APPL-positive membrane structures, now termed APPL endosomes, show only limited colocalization with EEA1 and are presently considered a distinct subpopulation of early endosomes. According to the current knowledge, both APPL proteins localize to the same structures (15) and thus can be interchangeably used as markers of APPL endosomes. However, to date no other specific markers of this compartment have been described. Moreover, APPL endosomes are usually localized in cells more peripherally than the canonical early endosomes, accumulating preferentially underneath the plasma membrane (15). This localization might reflect interactions between APPL endosomes and the elements of the cell cortex such as actin filaments; however, no such associations have been reported so far.

There is accumulating evidence suggesting that in addition to cargo trafficking, endosomes play a pivotal role in intracellular signal transduction. The canonical EEA1-positive early endosomes are involved in signaling processes initiated by various extracellular ligands (see Refs. 21–23 for review). Signaling functions have also been demonstrated for APPL endosomes which mediate Akt-dependent cell survival in zebrafish development (24) and activation of Akt and MAPK downstream of epidermal growth factor (EGF) or nerve growth factor (NGF) in mammalian cells (25,26). Moreover, APPL proteins themselves are involved in various signaling pathways, including signal transduction downstream of adiponectin (27), follicle-stimulating hormone (28), netrin-1 receptor DCC (29) or Wnt ligands (30). These findings raise a possibility that a signaling role of APPL endosomes extends into multiple pathways and ligand–receptor systems which merits attempts to define this compartment in more detail.

In this work we employed membrane fractionation methods to characterize APPL endosomes biochemically. In search for other potential markers of APPL endosomes we identified Annexin A2 as a protein co-fractionating with this compartment and interacting with both APPL1 and APPL2. Importantly, Annexin A2 appears to mediate APPL2 recruitment to the endosomal membranes which represents a novel role of Annexin A2 within the endocytic pathway.

## Results

### APPL endosomes are heterogeneous membrane structures of various densities

**Figure 1:**
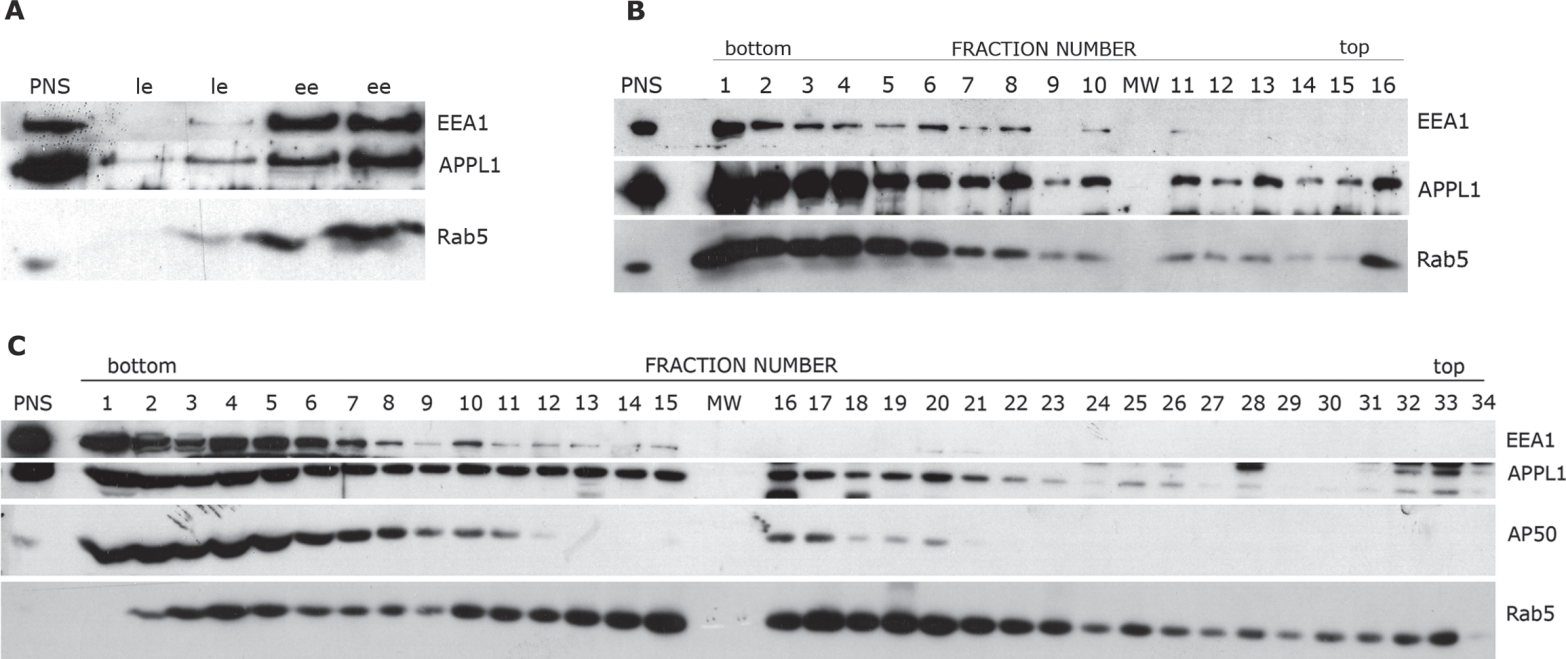
APPL endosomes are a heterogeneous population which exhibits a different fractionation pattern than the canonical early endosomes in the continuous density gradients PNSs extracted from S-HeLa cells were subjected to centrifugation in flotation gradients of sucrose (A and B) or OptiPrep (C). A) PNS sample adjusted to the final sucrose concentration 40.6% (w/w) was loaded at the bottom of a centrifuge tube and overlaid with step sucrose gradient (35, 25, 8.5%; all concentrations w/w). After centrifugation, fractions enriched in early endosomes (ee) and late endosomes (le) were collected from 35–25 and 25–8.5% interphase, respectively, and resolved on 10% SDS–PAGE followed by immunoblotting for EEA1, APPL1 and Rab5. Fractions from two independent gradients of the same PNS sample are shown. B) PNS sample was adjusted to the final sucrose concentration, 40.6% (w/w), and underloaded at the bottom of a 10–40% (w/w) continuous sucrose gradient. After centrifugation, 16 fractions of equal volume (800 µL each) were collected, pelleted, resolved on 10% SDS–PAGE and immunoblotted for EEA1, APPL1 and Rab5. C) PNS sample adjusted to the final OptiPrep concentration 40.6% (w/v) was underloaded at the bottom of a 5–20% (w/v) continuous OptiPrep gradient. Thirty-four fractions of equal volume (350 µL each) were collected, pelleted, resolved on the gradient (6–15%) SDS–PAGE and immunoblotted for EEA1, APPL1, Rab5 and AP50. In (A) and (B), 1.5 µL PNS (0.3% of the gradient input sample) was loaded as a control. In (C), 1 µL PNS (0.2% of the gradient input sample) was loaded as a control. MW, lane with a molecular weight marker.

To characterize the biochemical properties of APPL endosomes we investigated their distribution in density gradients in comparison to the canonical early endosomes harboring EEA1 (hereinafter referred to as EEA1 endosomes). Density gradient ultracentrifugation of cellular lysates is commonly used for the isolation and characterization of membrane-bound organelles. We decided to use floatation gradients, as they are superior over the sedimentation ones because membranes isolated by floatation are free from cytoplasmic contaminants or aggregates which can be present in membranes obtained by pelleting. In a classical method used for *in vitro* endosome fusion assays, functional populations enriched in either early or late endosomes are isolated from a post-nuclear supernatant (PNS) of cells by floatation during centrifugation in a step gradient of sucrose (4,31,32). We chose this method as a starting point and used PNS preparations from HeLa cells grown in suspension for which scaled-up fractionation procedures to obtain large quantities of endosomal material have been previously established (33). Using a step sucrose gradient (35–25–8.5%; all concentrations w/w), we could observe an enrichment of APPL1 protein in a fraction containing early endosomes as shown by the presence of Rab5 and EEA1 (Figure 1A). Although these results provided an independent demonstration that APPL endosomes represent a fraction of early endosomal compartments, no separation between APPL- and EEA1 endosomes could be observed by this technique. This was most likely because of insufficient resolution of a step sucrose gradient. To improve the resolution of endosome separation, we tested different ranges of continuous sucrose gradients (5–30, 20–40, 10–40%, all concentrations w/w; data not shown). The best resolution was obtained using a 10–40% sucrose gradient (corresponding to the densities of 1.0381–1.1765 g/mL), in which we could observe a partly distinct floatation pattern of EEA1- and APPL endosomes, providing a first indication for their non-identical physical properties (Figure 1B). In contrast to EEA1 endosomes, APPL1-containing vesicles exhibited a very broad migration through the whole gradient, suggesting their heterogeneity. As expected for an effector protein (APPL1) recruited to endosomes by a GTPase (Rab5), all fractions harboring APPL1 were also positive for Rab5.

To further improve the resolution of separation we employed OptiPrep (iodixanol) as a gradient medium, previously reported for the fractionation of endosomes (34–36). Its main advantage is low osmolarity which can minimize artifacts of the isolation procedure, in contrast to hyperosmolarity of concentrated sucrose solutions that may affect the properties of membrane-bound vesicles, e.g. causing their shrinking. Using a continuous 5–40% (w/v) OptiPrep floatation gradient (corresponding to the densities of 1.032–1.215 g/mL) we were again able to observe a broad migration pattern of APPL endosomes ranging from high- to low-density fractions (Figure 1C). Importantly, while high-density bottom fractions of the gradient (fractions 1–10 of densities 1.215–1.114 g/mL) contained both EEA1- and APPL endosomes, the latter could float to the fractions of much lower densities than EEA1 endosomes. In particular, we observed high accumulation of APPL endosomes in fractions 11–20 (densities 1.113–1.076 g/mL), with their traces present also in fractions 25–26 (1.057–1.054 g/mL) and 32–34 (1.040–1.033 g/mL). Such a broad gradient distribution points to a heterogeneous nature of APPL-harboring vesicles which seem to include membrane structures of different densities. At the same time, such biochemical heterogeneity largely precludes efficient isolation of fractions enriched in APPL endosomes through separation procedures based on physical properties such as density gradient centrifugation. Moreover, the migration of APPL endosomes was clearly distinct from that of CCVs characterized by the presence of the AP50 protein (AP-2 complex subunit µ) (37) (Figure 1C). Again, Rab5-positive vesicles were broadly distributed throughout all gradient fractions, consistent with the fact that several early endocytic structures (EEA1 endosomes, APPL endosomes, CCVs) are marked by this GTPase. Overall, the fractionation studies indicate that APPL endosomes represent a biochemically heterogeneous population, consisting of membrane structures of different densities. These data are in agreement with previous electron microscopy studies, documenting the presence of APPL1 on membrane profiles of various sizes and shapes (small vesicles, vacuoles or tubules) (15).

### Annexin A2 co-migrates with APPL endosomes on density gradients

To identify any further constituents residing on APPL endosomes, we attempted to establish an immunoisolation procedure for proteins bound to APPL1 on endosomes. However, APPL proteins are only peripherally associated with the endosomal membranes via active GTP-bound Rab5 molecules (15) and thus dissociate easily, particularly in cell lysates. Therefore, we employed chemical cross-linking using a cleavable, sulfhydryl-reactive homobifunctional cross-linker DPDPB to stabilize binding of proteins to the membranes (either in PNS or in gradient fractions enriched in early endosomes from HeLa cells) before the immunoisolation procedure. Such stabilized preparations were applied to Protein G Agarose with covalently bound anti-APPL1 antibodies or control rabbit immunoglobulin G (IgG). After washing and eluting, the resulting eluates were subjected to mass spectrometry sequencing. Although the efficiency of the procedure was low, we repeatedly identified Annexin A2 and the APPL1 protein itself in samples isolated on the anti-APPL1 column. Annexin A2 was the only protein reproducibly found in the immunoisolates obtained from both types of the starting material (PNS and endosome-enriched fractions).

**Figure 2:**
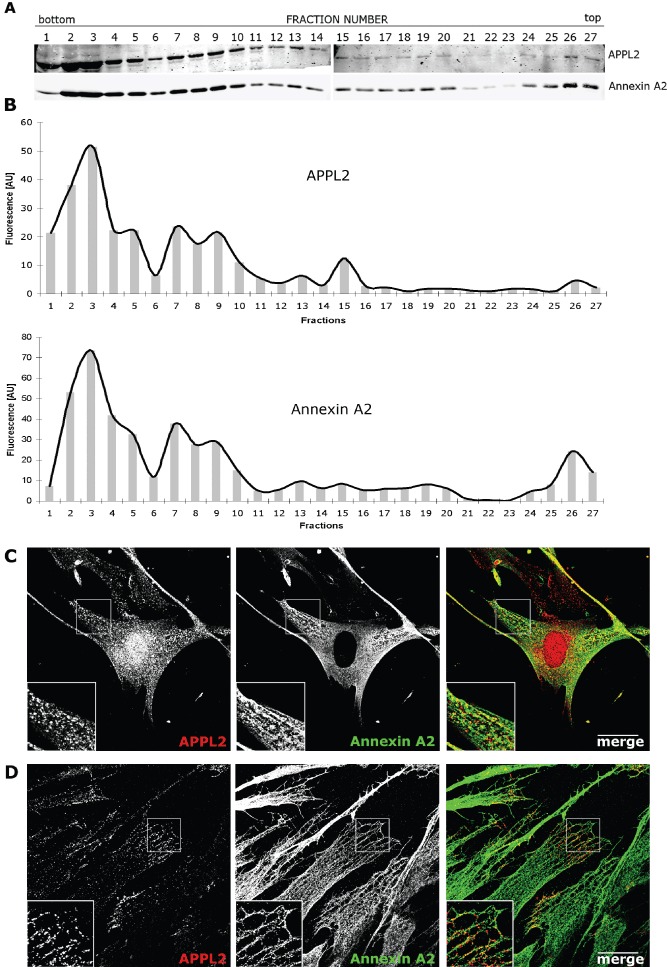
Annexin A2 and APPL2 show a highly similar migration pattern in a continuous OptiPrep gradient and colocalize with each other in cells A) PNS extracted from S-HeLa cells was adjusted to the final OptiPrep concentration, 40.6% (w/v), and underloaded at the bottom of a 5–20% (w/v) continuous OptiPrep gradient. After centrifugation, 27 fractions of equal volume (450 µL each) were collected, pelleted, resolved on the gradient (6–15%) SDS–PAGE and immunoblotted for APPL2 and Annexin A2. B) Western blot shown in A was subjected to densitometric analysis with Odyssey Infrared Imaging System. Fluorescence intensities of APPL2 and Annexin A2 bands in each fraction are expressed in arbitrary units (AU). C and D) Colocalization analysis of APPL2 and Annexin A2 in CCD-1070SK cells. In (C), cells were fixed, permeabilized and immunostained with antibodies against APPL2 (red) and Annexin A2 (HH7 clone; green). In (D), cells were initially permeabilized as described in *Materials and Methods* in order to wash away the cytoplasmic fraction of Annexin A2, then fixed and immunostained as in D. Single confocal sections are shown in (C) and (D). Scale bar, 20 µm.

Annexin A2 is known to exhibit a broad distribution among endocytic compartments, including early endosomes (38–40), and therefore could not represent an exclusive marker of APPL endosomes. However, among its various functions, Annexin A2 is an important mediator of the interaction between endosomes and the actin cytoskeleton (38,39,41,42), and for this reason we found its possible association with APPL endosomes of particular interest. We therefore investigated the distribution of Annexin A2 in membrane fractions resulting from the centrifugation in an OptiPrep density gradient. Similar to APPL2, Annexin A2 exhibited a broad migration ranging from high- to low-density fractions (Figure 2A). Quantitative analysis of the gradient fractions by immunoblotting and infrared imaging demonstrated a complex distribution pattern, including a few local accumulation peaks, which was very similar for both APPL2 and Annexin A2 (Figure 2B). These data indicate that both proteins may be present on the same membrane structures. To test this concept, we investigated the localization of both proteins by immunofluorescence microscopy. Using a standard procedure of cell fixation followed by permeabilization, we could not unequivocally demonstrate a direct colocalization of APPL proteins and Annexin A2 on vesicles because of the strong cytoplasmic staining of Annexin A2 (Figure 2C). Nevertheless, even under such conditions, APPL endosomes were clearly surrounded by Annexin A2 staining or in apparent contact with Annexin-positive fibrous structures. To reduce cytoplasmic staining of Annexin A2, cells were first permeabilized before fixation and in this case we could observe a clear colocalization of APPL2-positive endosomes with Annexin A2-labeled filamentous structures (Figure 2D). These data argue that at least some Annexin A2 colocalizes with APPL2.

### Annexin A2 interacts with APPL1 and APPL2

**Figure 3:**
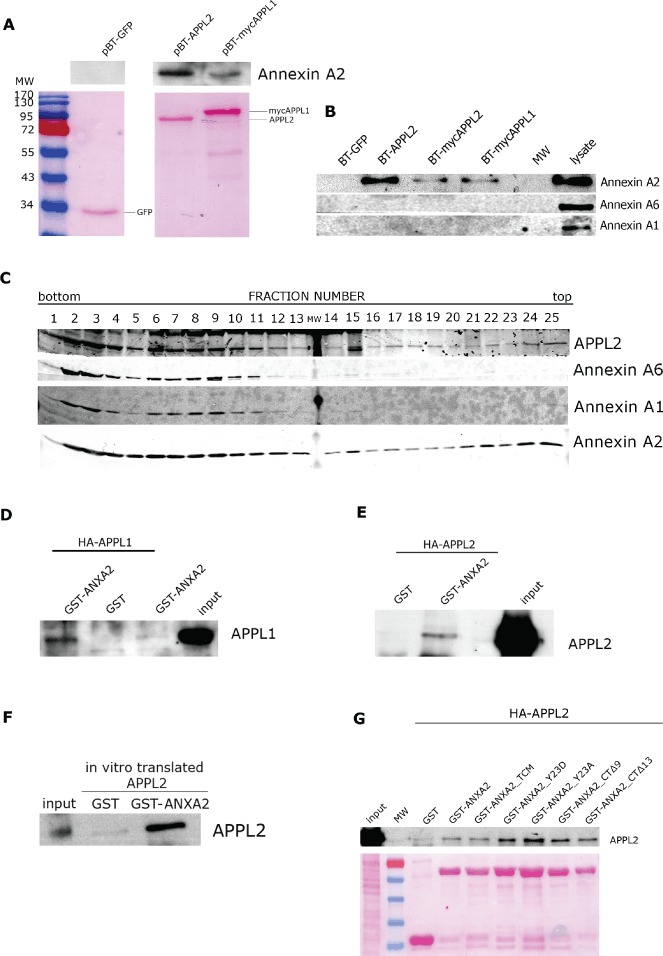
Annexin A2, but not Annexins A1 or A6, interacts with APPL proteins A and B) HEK293 cells were co-transfected with the plasmid encoding bacterial biotin-protein ligase (BirA) and one of the plasmids encoding GFP, APPL2 or APPL1 tagged with a BirA target sequence (pBT-GFP, pBT-APPL2, pBT-mycAPPL2 or pBT-mycAPPL1). Forty-eight hours after transfection, cells were lysed and affinity purification of APPL-interacting proteins was performed with streptavidine-conjugated magnetic beads. A) Binding of Annexin A2 to *in vivo* biotinylated APPL1 or APPL2 proteins is shown. Samples were resolved on 10% SDS–PAGE. Nitrocellulose membrane was stained with Ponceau S (lower panel) and immunoblotted for Annexin A2 (upper panel). B) Binding of Annexin A1 and Annexin A6 to *in vivo* biotinylated APPL1 or APPL2 proteins was tested. Samples were resolved on 10% SDS–PAGE and immunoblotted for Annexin A1 and Annexin A6, with Annexin A2 serving as a positive control. C) Migration of Annexins A1 and A6 in the OptiPrep density gradient. PNS sample adjusted to the final OptiPrep concentration 40.6% (w/v) was underloaded at the bottom of a 5–20% (w/v) continuous OptiPrep gradient. Twenty-five fractions of equal volume (500 µL each) were collected, pelleted, resolved on the gradient (6–15%) SDS–PAGE and immunoblotted for APPL2, Annexin A6, Annexin A1 and Annexin A2. D–F) GST pull-downs were performed with wild-type GST–Annexin A2 (GST–ANXA2) or GST alone with lysates from HEK293 cells overexpressing HA-APPL1 (D) and HA-APPL2 (E) or *in vitro* translated APPL2 (F). All samples were resolved on 8% SDS–PAGE and immunoblotted for APPL1 or APPL2, as indicated. As controls, 1 µL of cell lysates (1% of the input) was loaded in (D) and (E), and 1 µL *in vitro* translated protein (2% of the input) was loaded in F. G) GST pull-downs were performed with GST–Annexin A2 wild-type (GST–ANXA2) or mutants (TCM, Y23D, Y23A, CTΔ9, CTΔ13), or GST alone using lysates from HEK293 cells overexpressing HA-APPL2. All samples were resolved on 10% SDS–PAGE. Nitrocellulose membrane was stained with Ponceau S (lower panel) and immunoblotted for APPL2 (upper panel). As a control, 1 µL of cell lysate (1% of the input) was loaded. MW, lane with a molecular weight marker.

As Annexin A2 was a major protein present in APPL1 immunoisolates and co-migrated with APPL2 on density gradients, we further tested whether APPL proteins and Annexin A2 could interact. Therefore, we analyzed APPL-binding proteins using an *in vivo* biotinylation and affinity purification procedure. HEK293 cells were co-transfected with two vectors, one encoding a bacterial biotin ligase BirA and the other harboring APPL1 or APPL2 tagged with a BirA target sequence which undergoes biotinylation *in vivo*(43). Biotinylated green fluorescent protein (GFP) served as a specificity control. Proteins bound to biotinylated APPL1, APPL2 or GFP were isolated with streptavidine-conjugated beads and analyzed for the presence of Annexin A2 by western blotting. Strikingly, Annexin A2 was clearly retained on the beads containing either APPL1 or APPL2, the latter binding seemingly more Annexin A2 (Figure 3A). No association of Annexin A2 with control biotinylated GFP was detected. The presence of Annexin A2-derived peptides among APPL1- or APPL2-bound proteins was additionally confirmed by mass spectrometry analysis (data not shown). To test the specificity of Annexin A2 binding, we also tested other Annexins implicated in various endocytic steps, such as A1 and A6 (44). No interactions of Annexins A1 or A6 with APPL proteins could be detected by the same affinity purification procedure using biotinylated APPL1 or APPL2 (Figure 3B). Consistently, probing OptiPrep density gradient fractions for Annexins A1 and A6 revealed that both proteins were absent in the light gradient fractions enriched in Annexin A2 and APPL2 (Figure 3C).

To verify the interaction with APPL proteins by an independent method, glutathione S-transferase (GST)–Annexin A2 fusion protein was purified and used as a bait in a pull-down experiment with HEK293 cell lysates derived from cells overexpressing HA-APPL1 or HA-APPL2. As shown in Figure 3D,E, both APPL proteins were retained on GST–Annexin A2 beads, indicating that both APPL1 and APPL2 share the ability to interact with Annexin A2. To address the question of whether these interactions are direct, we performed an additional pull-down experiment using *in vitro* translated APPL2 instead of cell lysates. The results presented in Figure 3F demonstrate that APPL2 binds to the Annexin A2 in a direct manner. Although we could not convincingly show such direct binding for *in vitro* translated APPL1 because of its strong background binding to the beads, it is very likely that APPL1 also directly associates with Annexin A2 (particularly as we initially identified Annexin A2 in APPL1 immunoisolates, see above).

Finally, in order to obtain more insight into the nature of Annexin A2–APPL interactions, we tested several previously characterized Annexin A2 mutants for binding to APPL2 (Figure 3G). The following five mutants were expressed as GST fusion proteins: ‘*t*otal *C*a^2+^*m*inus’ [TCM; unable to bind calcium ions because of point mutations in type II and type III Ca^2+^-binding sites; (45)], two point mutants in Tyr23 residue which upon phosphorylation mediates binding of Annexin A2 to endosomes (46) [phosphorylation-deficient Y23A mutant which is not recruited to endosomes *in vivo*, and a phosphomimetic Y23D mutant capable of endosome binding which in addition affects actin dynamics in the cell (47)] and two C-terminal truncations of 9 or 13 amino acids (CTΔ9 or CTΔ13, respectively), unable to associate with F-actin (48). All of the tested mutants were able to interact with APPL2 in a GST pull down (Figure 3G). This indicates that the residues of Annexin A2 critical for binding calcium, endosomes or F-actin are not involved in its association with APPL2. It is therefore likely that these binding sites are independent and non-overlapping.

### Annexin A2 is essential for targeting APPL2 to endosomes and can compensate for Rab5 deficiency in mediating APPL membrane recruitment

**Figure 4:**
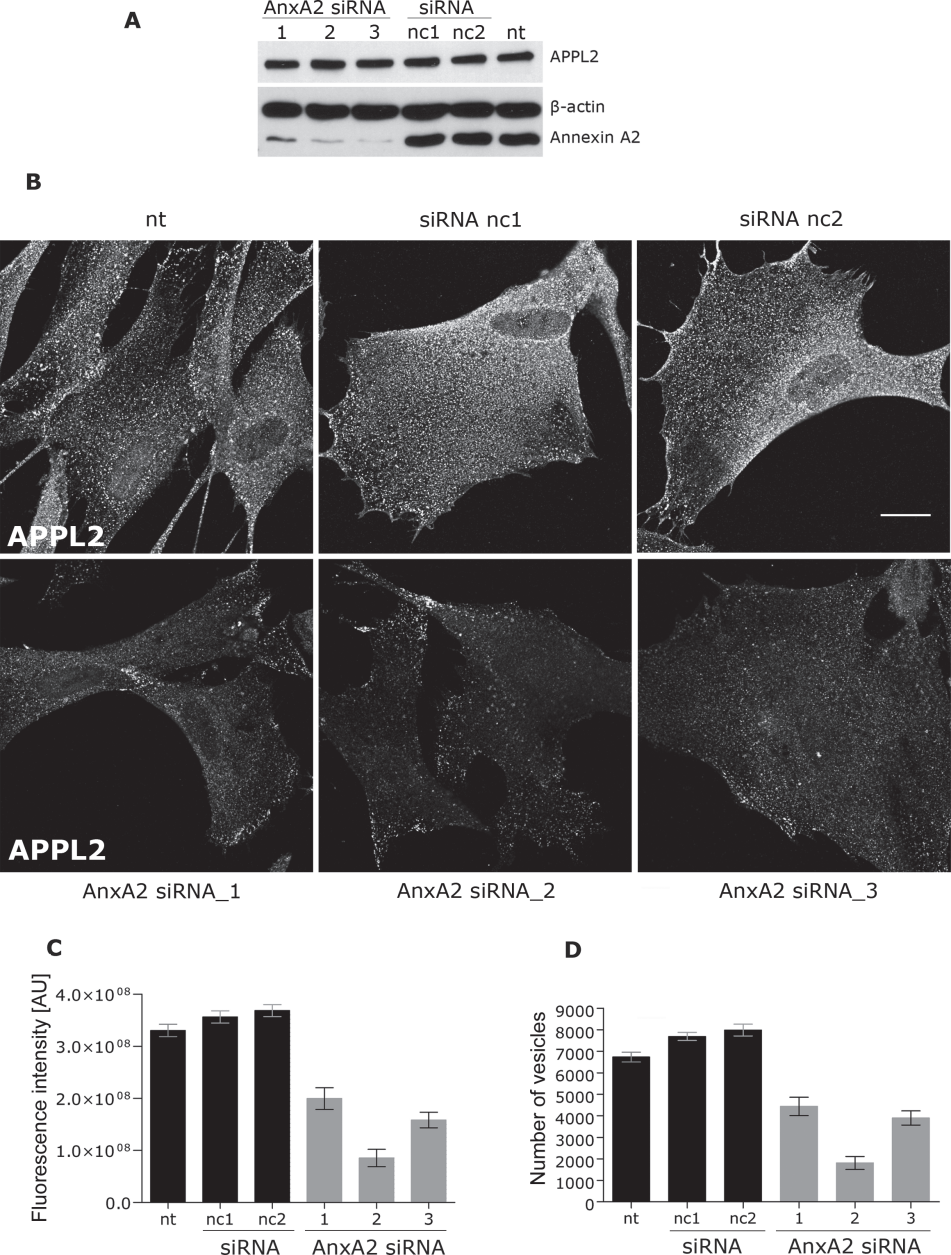

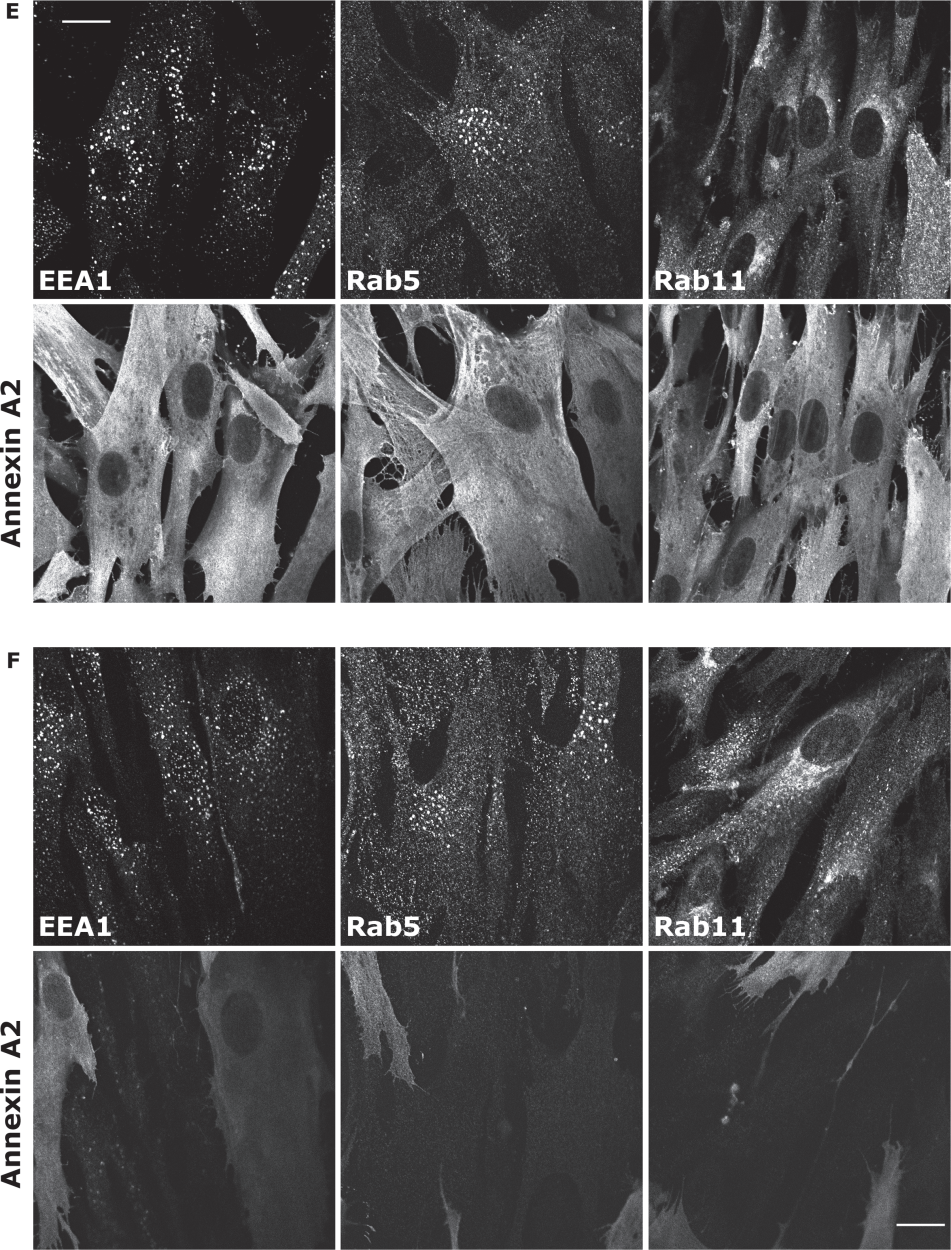
Annexin A2 knockdown causes solubilization of APPL2 from endosomes A–D) CCD-1070SK cells were transfected with three different siRNAs targeting Annexin A2 (AnxA2 siRNA_1-3) or two non-targeting negative controls (nc1, nc2), or were mock transfected (nt). A) Ninety-six hours after transfection, cells were lysed and the level of Annexin A2 was assessed. Samples were resolved on 8% SDS–PAGE and immunoblotted for APPL2 and Annexin A2, with β-actin as a loading control. B) Cells were fixed and immunostained with antibodies against APPL2. Single confocal images are shown. Scale bar, 20 µm. C and D) Total integral fluorescence intensity of APPL2 vesicles (C) and their average number (D) were quantified using MotionTracking software. At least 20 images of each variant were subjected to the analysis. The data are representative of three independent experiments. Error bars are SEM (standard error of the mean), fluorescence intensity is expressed in arbitrary units (AU). E and F) CCD-1070SK cells were transfected with non-targeting negative siRNA control nc2 (E) or with siRNA AnxA2 siRNA_3 targeting Annexin A2 (F), fixed and immunostained with antibodies against Annexin A2 and endosomal markers EEA1, Rab5 or Rab11, as indicated. Scale bar, 20 µm.

We wished to determine the functional importance of APPL–Annexin A2 interaction and hypothesized that it may be important for the morphology or cellular localization of APPL endosomes. To investigate this question, we chose human fibroblast cells CCD-1070SK which endogenously express high levels of Annexin A2 and APPL2, the latter used as a marker of APPL endosomes. We set out to test the effects of reduced Annexin A2 levels on APPL endosomes. Three independent siRNA duplexes were used to very efficiently silence the expression of Annexin A2 in CCD-1070SK fibroblasts, as shown in Figure 4A. Strikingly, when analyzed by confocal microscopy, these cells exhibited much lower staining of APPL2, as compared to cells transfected with control siRNAs (Figure 4B). All three duplexes targeting Annexin A2 showed similar effects. To quantify the observed changes, we employed image analysis software MotionTracking as a proven tool for measuring quantitative differences in parameters of objects detected by fluorescence microscopy (49,50). Such analysis demonstrated that the overall fluorescence of APPL2 detected in vesicular structures, categorized by the software as endosomes, is greatly reduced by Annexin A2 silencing (Figure 4C), although the total APPL2 levels in the cells remain unchanged (Figure 4A). These data indicate that APPL2 protein is displaced from the endosomal membranes and its cytoplasmic pool is likely in part removed during the immunostaining procedure and too disperse to be efficiently visualized. Consequently, because of the reduced membrane staining of APPL2, the number of detectable APPL endosomes is also decreased upon Annexin A2 knockdown (Figure 4D). These changes were specific for APPL2, as no solubilization of other endosomal markers such as EEA1, Rab5 or Rab11 was observed upon depletion of Annexin A2 (Figure 4E,F). Thus, our results allow concluding that the direct interaction between Annexin A2 and APPL2 appears to be required for the recruitment of APPL2 protein to the membrane of APPL endosomes.

**Figure 5:**
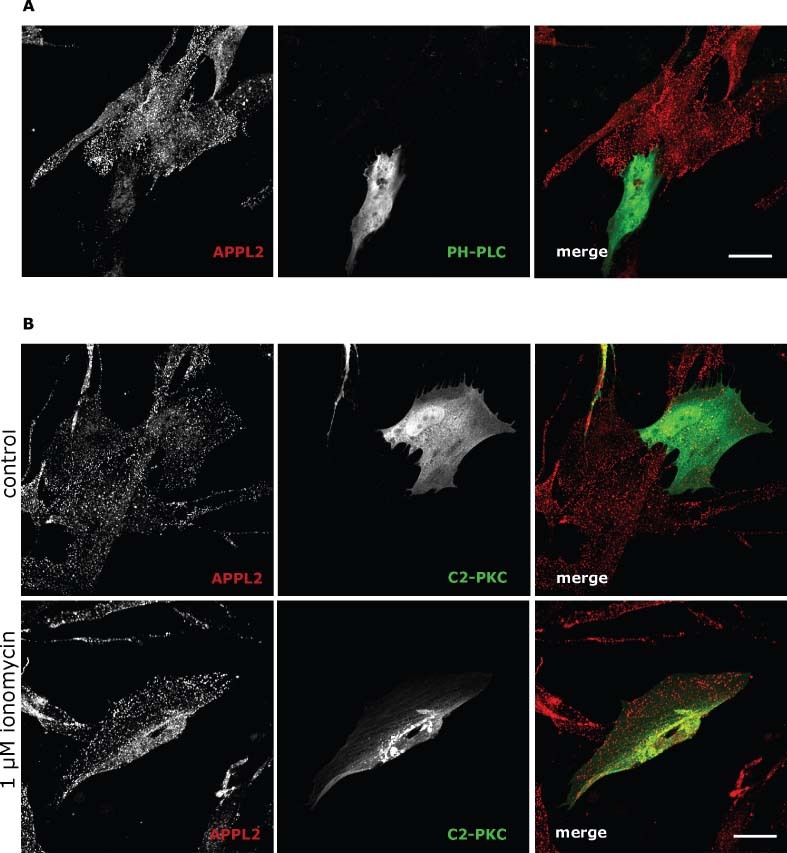
PI(4,5)P_2_ availability but not increased calcium concentration affects APPL2 localization to endosomes A) CCD-1070SK cells were transfected with the plasmid encoding the PH domain from PLCδ1 fused to GFP (PH-PLC) which specifically binds PI(4,5)P_2_. Cells were immunostained with antibodies against APPL2 (red) and GFP (green). Single confocal sections are shown. Scale bar, 35 µm. B) CCD-1070SK cells were transfected with the plasmid encoding the C2 domain from PKCγ fused to GFP (C2-PKC) which serves as an indicator of increased cytoplasmic calcium levels. Twenty-four hours upon transfection, cells were treated with 1 µm ionomycin (lower panel) for 1 min or left untreated (upper panel). Subsequently cells were fixed and immunostained with antibodies against APPL2 (red) and GFP (green). Single confocal sections are shown. Scale bar, 25 µm.

Annexin A2 is a calcium-binding protein which interacts specifically with phosphatidylinositol 4,5-bisphosphate [PI(4,5)P_2_] (45,51–53). To get further insight into Annexin A2-mediated endosomal recruitment of APPL2 we checked whether reducing the availability of PI(4,5)P_2_ or altering cytoplasmic calcium levels could affect membrane association of APPL2. To the first end, we overexpressed the PH domain of phospholipase Cδ (PH-PLC) which specifically binds to PI(4,5)P_2_ and at high levels should outcompete other PI(4,5)P_2_-binding proteins (54). Under these conditions, we observed a solubilization of APPL2 from endosomes (Figure 5A) which argues that PI(4,5)P_2_ levels are important for membrane association of APPL2. In contrast, increasing the cytoplasmic calcium concentration by ionomycin treatment did not affect an endosomal localization of APPL2 (Figure 5B). The efficiency of ionomycin action was controlled by observing calcium-induced clustering of Ca^2+^-binding C2 domain of protein kinase Cγ fused to GFP [C2-PKC] (55).

**Figure 6:**
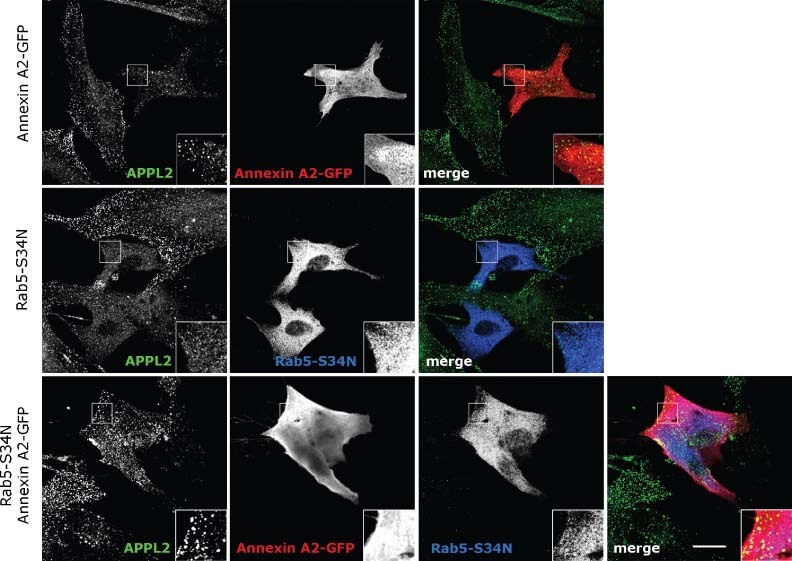
Overexpression of Annexin A2–GFP rescues the endosomal localization of APPL2 in cells transfected with the dominant-negative Rab5-S34N mutant CCD-1070SK cells were transfected with plasmids encoding Annexin A2–GFP, myc-tagged Rab5-S34N or both, as indicated. Cells were fixed and immunostained with antibodies against APPL2 (green), GFP (red) and myc (blue). Single confocal sections are shown. Scale bar, 20 µm.

Finally, we tested whether Annexin A2 co-operates with Rab5 in mediating membrane association of APPL proteins. Active Rab5 in a GTP-bound form is known to recruit APPL proteins to endosomes, while a GDP-locked, dominant-negative mutant Rab5-S34N solubilizes them from the membranes (15). We therefore checked the distribution of APPL2 upon overexpression of Annexin A2 in the presence of Rab5-S34N. Interestingly, high levels of Annexin A2 prevented loss of APPL2 from the endosomes caused by the Rab5 mutant (Figure 6). These data argue that Annexin A2 acts independently of Rab5 and can at least partly compensate for Rab5 deficiency in mediating membrane association of APPL proteins. Cumulatively, these results indicate that the presence of APPL proteins on endosomes is determined by at least two factors, such as active Rab5 and the levels of Annexin A2.

## Discussion

APPL endosomes were originally described in 2004, based mainly on light- and electron microscopy data which demonstrated significant segregation between APPL1 and a marker of canonical early endosomes, EEA1 (15). To date, only two specific markers of APPL endosomes are known, being APPL1 and APPL2 (of which often one is predominantly expressed in a given cell type; our unpublished observations). Rab5, which binds APPL proteins on APPL endosomes, also resides on other endocytic structures, such as the canonical EEA1 endosomes and CCVs, therefore cannot serve as a specific marker. In general, no biochemical studies of APPL endosomes have been undertaken and no other resident proteins reported.

Our studies employing various fractionation techniques demonstrate that (i) in step sucrose gradients APPL endosomes behave as early endosomes, as they partition with this compartment but not with late endosomes, and that (ii) APPL endosomes can be discriminated from the canonical EEA1-positive early endosomes by their partly different physical properties and a distinct migration pattern in continuous density gradients. Moreover, in contrast to the canonical EEA1-positive endosomes which appear to be much more homogeneous, APPL endosomes are biochemically heterogeneous and segregate into multiple gradient fractions of various densities. These results confirm the initial electron microscopy studies describing variable size and morphology of APPL-labeled membrane structures (15) although the molecular basis for such diversity of APPL endosomes remains unknown.

One of the main difficulties with detailed characterization of APPL endosomes, using either biochemical or microscopy methods, is lack of specific transmembrane markers. APPL proteins are both cytosolic and peripherally bound to the membranes (similar to Rab5). Any treatment causing solubilization of APPL proteins makes APPL endosomes impossible to distinguish from other Rab5-positive early endosomes. Therefore, it is so far not feasible to determine whether silencing of APPL proteins affects the functionality of the compartment. Similarly, while inhibition of clathrin-mediated endocytosis was shown to reduce the endosomal localization of APPL1 and was interpreted as the loss of APPL endosomes (16), it cannot be formally excluded that only membrane recruitment of APPL1 was abolished under such conditions, while the compartment *per se* was preserved. In our search for further markers of APPL endosomes, we attempted to circumvent the problem of APPL protein release by chemical cross-linking of either isolated membrane fractions or PNS before APPL1 immunoprecipitation. Although we failed to identify any transmembrane marker proteins, these experiments led to the identification of Annexin A2 as a component of APPL endosomes. While Annexin A2 is not an exclusive marker of APPL endosomes, it appears to interact with APPL proteins and regulate their membrane recruitment along with active Rab5. Importantly, high levels of Annexin A2 are sufficient to ensure membrane association of APPL2 even in the presence of inactive Rab5 mutant which alone causes dissociation of APPL proteins from endosomes. The fact that APPL membrane recruitment depends on two factors, active Rab5 and Annexin A2, is reminiscent of another Rab5 effector, EEA1 whose membrane localization is determined by the presence of active Rab5 and PI(3)P on endosomes. These two factors can also partly compensate for each other in recruiting EEA1 to early endosomes (6), similar to Rab5 and Annexin A2 in the case of APPL proteins.

Annexin A2 belongs to the annexin family of calcium- and phospholipid-binding proteins which exhibit a multitude of functions related to the organization and dynamics of membrane organelles (56). Among other compartments, Annexin A2 localizes to early endosomes and regulates their spatial distribution (38,39). Binding of Annexin A2 to early endosomes depends on cholesterol but is independent of calcium or p11/S100A10 ligand (46,57,58). Moreover, Annexin A2 directly interacts with actin filaments in a calcium-dependent manner (59) and binds to PI(4,5)P_2_(51–53). It regulates the formation of actin comets propelling macropinosomes (60) and is crucial for the nucleation of actin patches on early endosomes which are required for membrane remodeling during cargo transport toward late endosomes (42). It is thus tempting to speculate that Annexin A2 could link APPL endosomes to the actin cytoskeleton. Unfortunately, loss of APPL from the endosomal membranes upon knockdown of Annexin A2 precludes a direct test of such a hypothesis, as under these conditions APPL endosomes become largely undetectable. In many cell lines APPL endosomes are localized underneath the plasma membrane and more peripherally than the canonical early endosomes but the mechanisms of their anchoring to the cell cortex are not clear. Annexin A2 is known to govern the intracellular distribution of the canonical early endosomes (39) and it could have a similar function for APPL endosomes.

Finally, it has been shown that in fibroblasts of Lowe syndrome patients, Annexin A2 accumulates on actin comet tails which are constitutively formed on intracellular vesicles because of the increased levels of cellular PI(4,5)P_2_(61). This disease is caused by mutations in oculocerebrorenal syndrome of Lowe (OCRL), inositol 5-phosphatase (62) which preferentially acts on PI(4,5)P_2_ and phosphatidylinositol 3,4,5-trisphosphate [PI(3,4,5)P_3_]. Intriguingly, APPL1 is known to bind directly to OCRL (14,63,64). As Annexin A2 binds PI(4,5)P_2_, which is dephosphorylated by OCRL, it may suggest that the associations of APPL1 with Annexin A2 and with OCRL are mutually exclusive. Moreover, it may imply a temporal regulation of these interactions, where APPL binding to Annexin A2 would be important for the initial membrane recruitment of APPL and precede its association with OCRL which would lead to the local depletion of PI(4,5)P_2_ and thus Annexin A2 release. Although APPL2 does not bind OCRL directly (14), its indirect association with OCRL via heterodimers with APPL1 cannot be excluded.

In summary, in this work we characterized APPL endosomes as a biochemically discernible subpopulation of early endosomes and identified Annexin A2 as one of its components. Importantly, Annexin A2 appears to be required for endosomal localization of APPL2, acting along with Rab5. These data add yet another role to the spectrum of endocytic functions of Annexin A2, as a determinant of APPL membrane recruitment.

## Materials and Methods

### Cell culture and transfection

S-HeLa cells were grown in suspension in S-MEM (Spinner Modification of Minimum Essential Medium Eagle) supplemented with 10% newborn calf serum, 2 mml-glutamine, MEM Non-essential Amino Acid Solution (Sigma-Aldrich), 100 U/mL penicillin and 100 µg/mL streptomycin. CCD-1070SK and HEK293 cells were grown in MEM and DMEM, respectively, both supplemented with 10% fetal calf serum, 2 mml-glutamine, 100 U/mL penicillin and 100 µg/mL streptomycin under 5% CO_2_. For transfection of plasmids Lipofectamine-2000 (Invitrogen) was used according to the manufacturer's instructions. Ionomycin (Sigma-Aldrich) was used at 1 µm concentration.

### Antibodies and western blot quantitative analysis

Polyclonal antibodies against APPL1, APPL2 and EEA1 were previously described (15,30). Mouse anti-Annexin A2 clone HH7 (40) was a kind gift from Dr. V. Gerke. The following antibodies were obtained from commercial sources: anti-Rab5 (Abcam); anti-Rab11 (Invitrogen); anti-β-actin (Sigma); anti-Annexin A2, anti-Annexin A1, anti-Annexin A6, anti-AP50 and anti-EEA1 (BD Biosciences); anti-myc (clone 9E10 from Abcam). Goat antibody against GFP was obtained from MPI Dresden. Horseradish peroxidase (HRP)-conjugated anti-mouse and anti-rabbit antibodies were from Jackson ImmunoResearch; Alexa 488-, 555- or 647-conjugated anti-mouse, anti-rabbit or anti-goat secondary antibodies were from Invitrogen; IRDye 800CW donkey anti-mouse and IRDye 680LT donkey anti-rabbit antibodies from LI-COR Biosciences. Fluorescence quantitative analyses of western blots were performed with Odyssey Infrared Imaging System 3.0 (Li-COR Biosciences).

### Plasmids

To obtain pGEX-AnnexinA2, Annexin A2 open reading frame (ORF) (clone IRALp962P0840Q obtained from http://www.imagenes-bio.de) was subcloned to pGEX-6P-1 (GE Healthcare), using the following primers: forward TACGGATCCTCTACTGTTCAC, reverse TACCTCGAGTCAGTCATCTCC. Single-point mutations were introduced into pGEX-AnnexinA2 using QuikChange site-directed mutagenesis (Stratagene) to generate the following mutants: Y23D, Y23A (46), CTΔ9 or CTΔ13 (48). Annexin A2 TCM mutant was subcloned to pGEX-6P-1 from pDS10-AII-TCM received from Dr. V. Gerke (45). Plasmids pcDNA3.1-APPL2, pcDNA3-HA-APPL1 and pcDNA3-HA-APPL2 were previously described (15,30). Plasmids for expression of BirA and of biotinylated GFP (pBT-GFP) were a kind gift of Dr. C. Hoogenraad. To obtain plasmids for expression of biotinylated APPL2 and myc-APPL1 (pBT-APPL2, pBT-mycAPPL1), the following pairs of primers were used for subcloning into pBT: APPL2 forward GTCGTCGACATGGAGCAGAAGCTGATCTCCGAGG and reverse GCGGGCGGCCGCTTATGCTTCTGATTCTCTCTTC; APPL1 forward GTCGTCGACATGCCCGCCGTGGACAAGCTCCTGC and reverse CCGCGCGGCCGCTTATGCTTCGGATTCTGCGCCTC. The plasmid GFP-pcDNA3-PKCgamma-C2 was provided by Dr. Tobias Meyer (Addgene plasmid 21215) (55). The plasmid en-coding the PH-PLC was previously described (65).

### Preparation of PNS

S-HeLa cells were grown at the density of 10^6^ cells/mL in a spinner flask in a total volume of 2.4 L. Cells were briefly centrifuged, and washed three times with PBS, followed by two washes in SIM buffer (250 mm sucrose, 3 mm imidazole, 1 mm MgCl_2_ pH 7.4). Cell pellets were resuspended in 2 volumes of SIM buffer with 1 mm DTT and protease inhibitors. Cells were broken by 10 passages through an ice-cold ball-bearing homogenizer (EMBL Heidelberg) with a 16-µm clearance. The resulting cell homogenates were spun for 20 min at 2000 ×***g*** at 4°C. Supernatant (PNS) was collected for further analysis.

### Fractionation of endosomes by ultracentrifugation in density gradients

Endosome fractionation on a step sucrose gradient was conducted essentially as previously described (66). In brief, PNS was adjusted to 40.6% (w/w) sucrose by mixing with 62% (w/w) sucrose solution and 1.1 mL of such mix was loaded at the bottom of 13.2 mL ultracentrifuge tube. It was overlaid with 4 mL 35% (w/w) sucrose in 3 mm imidazole pH 7.4, 4 mL 25% sucrose (w/w) in 3 mm imidazole pH 7.4 and the tube was filled with SIM buffer [8.5% (w/w) sucrose]. The loaded gradient was centrifuged in an SW 41 Ti rotor (Beckmann Coulter) at 100 000 ×***g*** for 6 h at 4°C. Early endosome fraction was collected with the peristaltic pump from the 25–35% interphase and the late endosome one from the 25% SIM interphase.

The continuous 10–40% sucrose gradient was made using a Gradient Master (BioComp Instruments, Inc.). The gradient was prepared freshly in a 13.2 mL tube. PNS was adjusted to 40.6% (w/w) sucrose by mixing with 62% sucrose solution and 1.1 mL of such mix was underloaded at the bottom of the gradient. The loaded gradient was centrifuged in an SW 41 Ti rotor at 100 000 ×***g*** for 6 h at 4°C. After ultracentrifugation, 16 fractions of equal volume (800 µL) were collected with the peristaltic pump, diluted in ice-cold PBS and pelleted by centrifugation (110 000 ×***g*** for 45 min), resolved on 10% SDS–PAGE and immunoblotted for the proteins of interest. The linearity of gradients was checked with a refractometer.

OptiPrep gradients were prepared from a ready-made solution of 60% iodixanol (w/v) in water, commercially available under the name of OptiPrep (Axis-Shield; density: 1.32 ± 0.001 g/mL; osmolarity: 170 ± 15 mOsm). The working iodixanol solutions (5 and 20%, w/v) for gradients were prepared by two subsequent dilution steps as follows. First, to prepare a 40% (w/v) solution, 2 volumes of 60% iodixanol were mixed with 1 volume of the OptiPrep dilution buffer (235 mm KCl, 12 mm MgCl_2_, 25 mm CaCl_2_, 30 mm EGTA, 150 mm HEPES–NaOH pH 7.0). Second, to obtain 5 and 20% solutions, 40% iodixanol (w/v) was diluted with the working solution dilution buffer (78 mm KCl, 4 mm MgCl_2_, 8.4 mm CaCl_2_, 10 mm EGTA, 50 mm HEPES–NaOH pH 7.0).

The continuous 5–40% OptiPrep gradient was prepared freshly in a 13.2 mL tube. PNS was adjusted to 40.6% (w/v) OptiPrep by mixing with 60% OptiPrep and 1.1 mL of such mix was underloaded at the bottom of 5–20% gradient pre-made using a Gradient Master. The loaded gradient was centrifuged in an SW 41 Ti rotor at 100 000 ×***g*** for 20 h at 4°C. After ultracentrifugation, fractions of equal volume were collected, diluted in ice-cold PBS and precipitated by centrifugation (at 110 000 ×***g*** for 45 min), resolved on SDS–PAGE and immunoblotted for the proteins of interest. The linearity of gradients was checked with a refractometer.

Early endosome-enriched fraction, as a starting material for immunoisolation, was obtained from the OptiPrep step gradient (40–18–5%). PNS was adjusted to 40% (w/v) OptiPrep by mixing with 60% (w/v) OptiPrep solution, and 1.1 mL of such mix was loaded at the bottom of 13.2 mL ultracentrifuge tube. It was overlaid with 8 mL 18% (w/v) OptiPrep, and the tube was filled with 5% (w/w) OptiPrep. The loaded gradient was centrifuged in an SW 41 Ti rotor (Beckmann Coulter) at 100 000 ×***g*** for 6 h at 4°C. Early endosome fraction was collected with the peristaltic pump from the 5–18% interphase.

### Cross-linking of membrane-bound proteins and immunoisolation of APPL-associated complexes

1,4-Di-(3′-[2′pyridyldithio]-propionamido) butane (DPDPB) was used for the cross-linking of PNS or a gradient fraction enriched in early endosomes (collected from the 5–18% interphase of a step Optiprep gradient). Four hundred microliters of PNS adjusted to a final volume of 1 mL with 5% (w/v) OptiPrep or 1 mL of an OptiPrep gradient fraction enriched in early endosomes was mixed with DPDPB to a final concentration of 5 mm. After 30 min of incubation at 30°C, excess of DPDPB was quenched with 50 mml-cystein for 45 min at 4°C.

To prepare a resin with covalently bound antibodies, 80 µL of Protein G Agarose (Roche) was equilibrated with 10 mm Tris–HCl pH 8.0 and mixed with 4 µL of rabbit anti-APPL1 antibodies or unspecific rabbit immunoglobulins. After 90 min of incubation at room temperature and a brief centrifugation the resin was washed with 200 mm Na_2_B_4_O_7_ pH 9.0, and incubated for 30 min with 20 mm dimethyl pimelimidate (DMP) cross-linker in 200 mm Na_2_B_4_O_7_ pH 9.0. Afterwards the resin was washed with 200 mm ethanolamine pH 8.0 to quench the excess of DMP and incubated with 200 mm ethanolamine pH 8.0 for 2 h at room temperature, followed by a series of washes with PBS or PBS with 500 mm NaCl and finally equilibrated with immunoisolation buffer [150 mm NaCl, 1 mm EGTA, 1 mm ethylenediaminetetraacetic acid (EDTA), 1% Triton-X-100, 10% glycerol].

For immunoisolation, 80 µL of antibody-conjugated resin was mixed with approximately 220 µg of cross-linked proteins from PNS or early endosome-enriched gradient fractions, and incubated overnight at 4°C, followed by a series of 10 washes with the immunoisolation buffer. Proteins bound to the resin were eluted with 100 mm glycine pH 2.5 at room temperature. The eluates were mixed with Laemmli buffer and loaded on SDS–PAGE. Silver-stained bands were analyzed by mass spectrometry at the Institute of Biochemistry and Biophysics in Warsaw.

### GST pull-down and in vitro binding assays

The full-length GST–Annexin A2 fusion protein, its mutant versions or GST alone were expressed and purified according to the manufacturer's instructions (GE Healthcare). Isopropyl-1-thio-β-d-galactopyranoside (Sigma) at a concentration of 0.5 mm was used to induce protein expression. HEK293 cells transfected with pcDNA3-HA-APPL1 or pcDNA3-HA-APPL2 were lysed in ice-cold pull-down buffer (150 mm NaCl, 50 mm HEPES pH 7.4, 1 mm EDTA, 1 mmEGTA, 10% glycerol, 1% Triton-X-100 and protease inhibitors). The purified GST–Annexin A2 or GST alone bound to the glutathione-Sepharose 4B beads (GE Healthcare) were incubated overnight at 4°C with HEK293 lysates. After incubation, beads were washed with the pull-down buffer. Bound proteins were eluted with 10 mm glutathione in 50 mm Tris–HCl pH 8.0 for 15 min at 22°C. Eluates were resuspended in Laemmli buffer, resolved on SDS–PAGE and immunoblotted for the proteins of interest.

*In vitro* translation was carried out in a TNT coupled reticulocyte lysate using the Transcend Non-Radioactive Translation Detection System (Promega) according to the manufacturer's recommendations. *In vitro* translated protein was incubated overnight at 4°C with constant rotation with 11 µL of glutathione-Sepharose 4B beads carrying bound GST or GST–Annexin A2 fusion protein. After incubation beads were washed and eluted as above.

### In vivo biotinylation and affinity purification of APPL-binding proteins

For biotin–streptavidin pull-down assays HEK293 cells were transfected with the plasmid encoding bacterial biotin ligase (BirA) (43) and pBT-GFP, pBT-APPL2 or pBT-mycAPPL1, respectively. Forty-eight hours post-transfection, cells were lysed in a lysis buffer (150 mm KCl, 20 mm Tris–HCl pH 8.0, 1% Triton-X-100, protease inhibitors). Cell lysates were centrifuged at 20 800 ×***g*** for 15 min and the supernatants were incubated with Dynabeads M-280 streptavidin (Dynal; Invitrogen) for 45 min. Beads were separated by using a magnet (Dynal; Invitrogen) and washed five times in a lysis buffer. For protein elution, the beads were boiled in Laemmli buffer. Samples were resolved on 10% SDS–PAGE and immunoblotted with anti-Annexin A2 antibody (BD Biosciences).

### RNA interference

Three siRNA duplexes targeting human Annexin A2 (AnxA2 siRNA_1: 5′-GCAAGUCCCUGUACUAUUAtt-3′; AnxA2 siRNA_2: 5′-GAACUUGCAUCAGCACUGAtt-3′; AnxA2 siRNA_3: 5′-CCAGCUUGCGAAUAACAGUtt-3′), as well as two non-targeting negative control duplexes were obtained from Ambion. CCD-1070SK cells were transfected in 24-well dishes with Lipofectamine LTX (Invitrogen) according to the manufacturer's protocols. Analyses were performed 96 h after transfection.

### Immunofluorescence and quantitative image analysis

CCD-1070SK cells were plated on 12-mm coverslips in 24-well plates in 500 µL DMEM medium with 10% serum, and transfected if appropriate. They were fixed with 3% paraformaldehyde, permeabilized with 0.1% (w/v) saponin and processed for immunofluorescence with appropriate primary and secondary antibodies. Alternatively, cells were permeabilized with 0.1% Triton-X-100 prior to fixation as described previously (67). For quantitative analysis of APPL2 signal, at least 20 images (12 bit pixel depth) per condition were taken using a confocal microscope (Leica TCS SP2 with AOBS) with a 63×/1.4 numerical aperture (NA) oil immersion objective, 200 Hz speed and 1024 × 1024 pixel resolution. Images were exported as TIFF files directly into the MotionTracking/Kalaimoscope software (http://www.kalaimoscope.com) (49,50). Two morphometric parameters of APPL endosomes were calculated: (i) the total fluorescence of a fluorophore (APPL2) detected in all vesicles, defined as the total integral vesicle intensity and expressed in arbitrary units (AU), and (ii) the number of vesicles. Both parameters were calculated per masked area of each image.
